# Ultrasonic vs Drill Implant Site Preparation: Post-Operative Pain Measurement Through VAS, Swelling and Crestal Bone Remodeling: A Randomized Clinical Study

**DOI:** 10.3390/ma11122516

**Published:** 2018-12-11

**Authors:** Antonio Scarano, Francesco Carinci, Felice Lorusso, Felice Festa, Lorenzo Bevilacqua, Pablo Santos de Oliveira, Michele Maglione

**Affiliations:** 1 Department of Medical, Oral and Biotechnological Sciences and CeSi Met, University of Chieti-Pescara, 66100 Chieti, Italy; drlorussofelice@gmail.com (F.L.); felice.festa@unich.it (F.F.); 2 Department of Maxillofacial Surgery, University of Ferrara, 44121, Ferrara, Italy; francesco.carinci@unife.it; 3 Department of Medical Sciences, University of Trieste, 34127, Trieste, Italy; l.bevilacqua@fmc.units.it (L.B.); michelemaglionets@gmail.com (M.M.); 4 Department of Oral Implantology, Dental Research Division, College Ingà, UNINGÁ, Cachoeiro de Itapemirim 29312, Brazil; psoliveiraodonto@yahoo.com.br

**Keywords:** piezosurgery, ultrasonic surgery, implant bed preparation, microcracks, pain, crestal bone resorption

## Abstract

**Background**: Piezosurgery is a surgical procedure that is able to perform osteotomies by a micrometric and selective cut of the bone tissue. The objective of this investigation was to evaluate two different techniques; an ultrasonic device, and a drill approach for implant site preparation. **Methods**: A total of fifty patients were recruited for the randomized clinical trial to receive dental implants for fixed prosthetic restoration in the posterior mandible and were allotted to two groups. In Group A the implant site was prepared following a drilling technique, while in Group B the implant site was prepared using an ultrasonic device; moreover, the operative duration was recorded. Postoperative pain and swelling were evaluated at 1, 2, 4, and 6 days. The crestal bone resorption was measured at 3 months from implant placement by a three-dimensional tomography evaluation. **Results**: The findings suggest that osteotomies performed by an ultrasonic device cause less pain and swelling. On the other hand, the piezoelectric preparation was characterized by a significative increase in the operative time. No statistical differences in crestal bone resorption were evident in the two different approaches. **Conclusions**: According to the outcome of the study, ultrasonic implant bed preparation can be used with success in implantology and could be considered a suitable alternative to traditional drilling techniques for dental fixture placement.

## 1. Introduction

Nowadays, osseointegrated implants have very high clinical long-term predictability [1,2,3,4], and in recent years there has been great changes regarding their surface properties. New materials have been studied and proposed to increase the manageability of positioning and prognosis of dental implants. The response of the tissues to the application of biomaterials is determined and influenced by the surface properties [5,6]. In fact, it has been demonstrated that the features of the fixture surface in terms of nano, micro topography, and physicochemical properties are key factors in achieving dental implant osseointegration, in particular under the histological profile, aiming at biological and morphological compatibilities [7,8].

The achievement of primary stability of the fixture, bone tissue density and quality, the implant micro-design, surface properties, are all necessary factors in the successful osseointegration of dental implants during surgical procedures [9,10]. The healing of the bone tissue surrounding dental implants is a result of complex phenomena. The process requires the differentiation and proliferation of pre-osteoblasts into osteoblasts, through the induction of precursor cells by the periosteum and the endosteum, which evolves into the synthesis and subsequent maturation of the osteoid matrix and the promotion of the bone-fixture interface [11]. Thus, the success of the procedure for dental implant placement is correlated with the ability to obtain primary healing [12]. Moreover, a minimally traumatic approach to the implant bone bed is an important key factor [13].

Atraumatic preparation of the surgical site is essential for correct connection between the dental fixture and bone tissue, because it influences the induction and progress of the subsequent bone healing process [14]. For this reason, improving the bone cutting techniques and osteotomies in oral surgery and surgical medicine assumes a determining role in reducing mechanical and thermal tissue trauma [15]. Piezosurgery is a technique that enables accurate and safe osteotomy lines through micrometric and selective bone cutting, based on the generation of ultrasonic vibrations [16]. Indeed, the properties and design of the cutting device, the drilling speed and pressure, osteotomy shape and procedure pattern, and finally, the use of supplemental irrigation can significantly influence bone tissue damage. Though the findings are conflicting and based on different studies, it has been reported in the literature that the choice of an ultrasonic approach could be correlated with decreased postoperative pain. This evidence is related to reduced thermal diffusion to the surrounding tissues, close to the sympathetic chain [17].

Nevertheless, the conventional drill approach represents the gold standard for implant bed preparation and the clinical protocol is relatively manageable. In the literature, histological and biomechanical data on the osseointegration obtained by piezoelectric osteotomy are scarce [18,19].

The ultrasonic surgical approach for osteotomy and osteoplasty procedures has significant effects on the ratio of repair and remodeling processes of the bone tissues [20,21], on bone chip morphology, and on the proliferation and differentiation of cells deriving from grafts collected from intraoral donor regions [22]. In the literature, it has been demonstrated that piezoelectric osteotomy also has important effects on wound healing, promoting angiogenesis processes [23], and the protection of local soft tissue structures around the site and the precision of the osteotomy [24,25].

The objective of the present study was to evaluate and compare two different implant site preparation techniques using piezoelectric surgery vs conventional drilling, through the evaluation of pain, swelling and crestal bone remodeling.

## 2. Results

### 2.1. Group A (Drill)

#### 2.1.1. Clinical Observation

Macroscopic observation showed irregular and jagged margins of the osteotomical line in the drill prepared implant sites, producing visible bone chips.

All study patients successfully completed the treatment and the healing process was uneventful. No neurosensory disturbances were recorded in relation to the procedures. Twenty-four dental fixtures appeared stable and well osseointegrated at the end of the experimental period, and no evidence of inflammation foci were recorded in relation to the healing phase, only one implant showed peri-implant radiolucence and was removed.

#### 2.1.2. Pain and Swelling Intensity Evaluation

The level of intensity of the pain was collected at day 1 after surgery with a score of 16.33 ± 4.12 mm and was classified according to Visual Analog Scale (VAS) scores as mild pain, while at two days it was 19.22 ± 2.22 mm. The pain intensity at four days was significantly decreased, with a value of 0.92 ± 0.3 mm.

After six days, the pain intensity decreased to 0.11± 0.02 mm.

The swelling score was 1.9 ± 0.8 at first day, 1.2 ± 0.4 at the second and was significantly reduced after four days at 0.36 ± 0.31.

**Figure 1:**
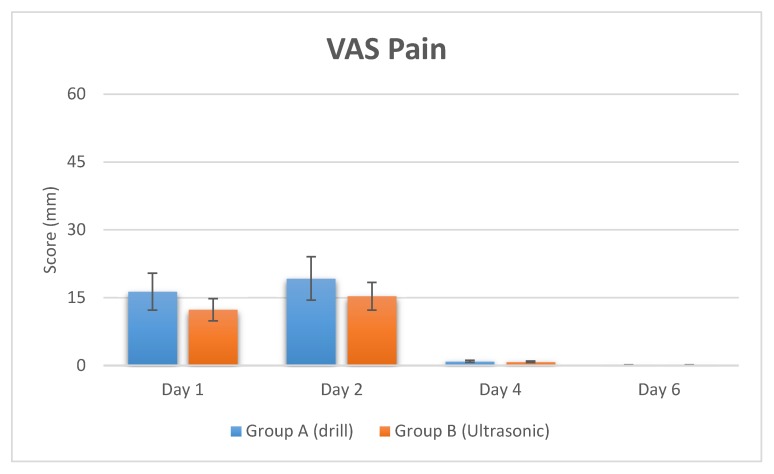
Pain rate of patients in post-operative times recorded by VAS Score.

**Figure 2:**
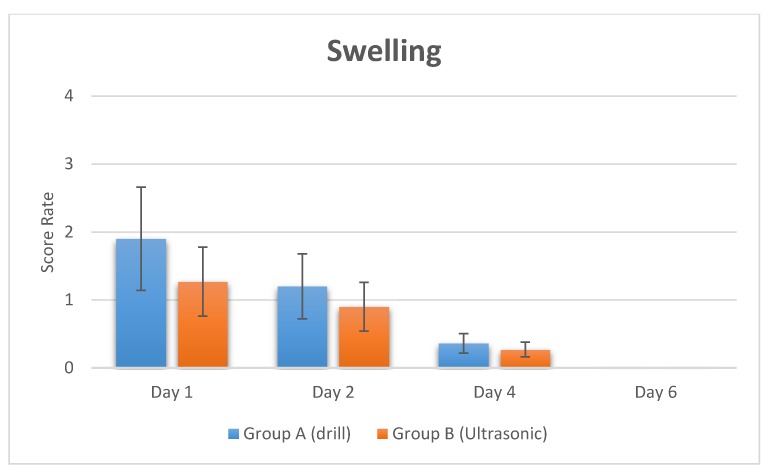
Swelling in post-operative times recorded by swelling score after implant treatment by Verbal rating scales (VRS).

No evidence of swelling was recorded on day 6 after the surgery (Figure 1 and Figure 2, and Table 1).

#### 2.1.3. Operative Duration and Crestal Bone Level Measurement

**Figure 3:**
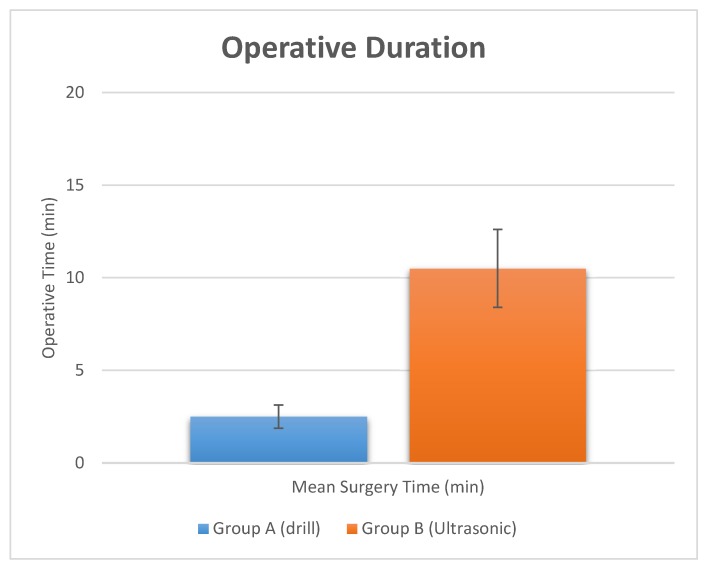
Operative surgery duration evaluated for the two study groups.

**Figure 4:**
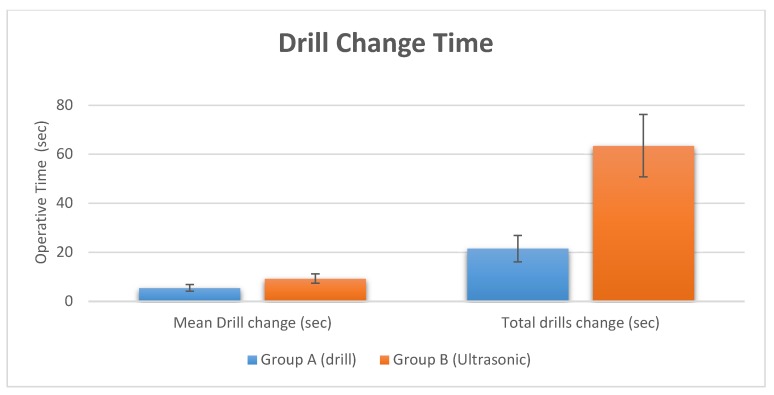
Drill change records of the two different site preparation techniques.

**Figure 5:**
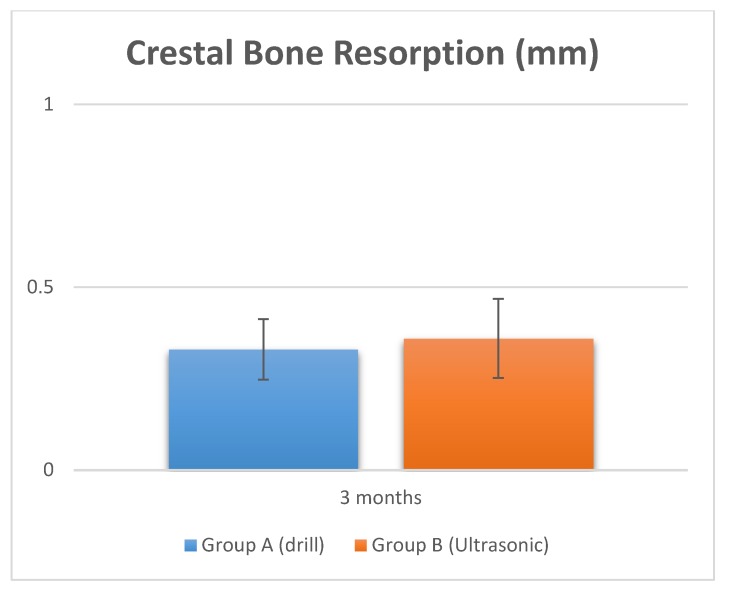
Crestal bone resorption at 3 months healing.

The surgical time required to perform and complete the implant site preparation procedures, excluding the time needed for changing the drills, was 2.5 ± 0.34 min. The time required for changing each drill was 5.5 ± 2.1 s for a total 21.5 ± 2.1 s (Figure 3 and Figure 4, and Table 2). Crestal bone resorption was 0.03 ± 0.001 mm after 3 months of healing (Figure 5 and Table 2).

### 2.2. Group B (Ultrasonic Instruments)

#### 2.2.1. Clinical Observations

Macroscopic evaluations demonstrated the regular and homogeneous shape of the osteotomic margins with no evidence of bone chips detached from the site walls.

No neurosensory disturbances were recorded, and all the patients successfully completed the study and the healing process was uneventful. At the end of the experimental period, a total of twenty-four dental fixtures appeared stable and well osseointegrated, and during the healing phase no evidence of inflammation foci was found. Only one implant showed a larger bone resorption and was removed.

#### 2.2.2. Pain and Swelling Intensity Evaluation.

At day 1 after the surgery the pain was 12.33 ± 2.32 mm and was classified as mild pain while at two days it was 15.32 ± 3.34 mm. At four days the pain intensity recorded was 0.82 ± 0.01 mm.

After six days, the pain intensity had disappeared, being 0.1± 0.01 mm.

The swelling score was 1.27 ± 0.6 on the first day and 0.9 ± 0.58 on the second day. The swelling was significantly reduced after four days, being 0.27 ± 0.14. No evidence of swelling was reported on day 6 after the surgery (Figure 1 and Figure 2, and Table 1).

#### 2.2.3. Operative Duration and Crestal Bone Level Measurement

The surgical time required to complete the implant bed preparation procedures, excluding time needed for changing the tips, was 10.5 ± 3.1 min. The time required for changing each tip was 9.3 ± 3.2 s for a total 63.5 ± 2.1 s (Figure 3 and Figure 4, and Table 2). Crestal bone resorption was 0.036 ± 0.01 mm after 3 months of healing (Figure 5 and Table 2).

## 3. Discussion

The aim of the present investigation was to study the post-operative pain of surgery and implants positioned in sites prepared with two different techniques: using conventional drills and an ultrasonic approach. The investigators hypothesized that implant bed preparation with ultrasonic tips may offer good clinical results with reduced swelling and pain. The study design provides only posterior mandible sites because in this area there is a cortical bone and for reduced variables. In this study model, each patient received only two dental fixtures in order to decrease the number of experimental variables. Furthermore, in the posterior mandible region the risk of damaging the alveolar nerve is greater [26], in fact, implant bed preparation with a piezoelectric device reduces the possible damage to the inferior alveolar nerves [27,28].

The outcome of this clinical study demonstrates the great effectiveness of the ultrasonic technique in performing implant preparation, with a significant decrease of postoperative pain and swelling. On the other hand, the study shows that these advantages are related to a significant increase in operating time in the piezoelectric surgical approach. Less pain and swelling suggest that the bone contained vital cells, alkaline phosphatase and osteoblast activity, which promotes the healing of the implant site prepared with the ultrasonic method. Therefore, the study results suggest that there is increased clinical healing related to the osteotomy performed by the piezoelectric approach if compared with standard drill preparation; this is consistent with clinical studies [20] and histological studies in rats [29]. Also, Di Alberti et al. [30], reported an increased mean value of bone density around dental fixtures positioned in bone sites treated by an ultrasonic approach if compared to bone sites prepared with the standard drilling technique.

Today, most clinical surgery uses a conventional, easy rotary procedure for site preparation. However, this approach does not provide selective cutting action on the bone and therefore, it will probably damage the surrounding tissues encountered, such as soft tissues, nerve structures and blood vessels.

Initial primary stability is an important key factor for successful osseointegration of the dental implant [31]. Besides surface properties, and sufficient quantity and quality of the native bone volume, the surgical technique and protocol have a key role in providing a predictable and long-term bone to implant contact. Mechanical and thermal trauma generated in the tissues in relation to the surgical site preparation, deeply influence the progress of the bone healing and processes of osseointegration [14,32]. The influence of technical settings, like the speed of the drilling procedure and pressure, as well as mechanical bone condensing techniques, are reported in the scientific literature [33,34].

The use of a piezoelectric approach to perform bone cutting has been related to rapid new bone formation, whilst in approaches that used rotating instruments and drills, the newly formed tissue showed a less advanced level of maturation related to the healing process, where the central part of the bone osteotomies appeared to be filled by non-mineralized fibrovascular tissue [35]. The histological evidence of live osteocyte cells characterized by physiological dimensions and morphology on the cut margin surfaces, probably indicates the reduced trauma of the piezoelectric osteotomic cut with a complete absence of harmful side effects [35]. Also, no differences were evident on the cancellous bone. This phenomenon reduces evidence of inflammatory processes, and produces faster bone remodeling with an increase in the levels of BMP-4 and TGF-b2 molecules [18].

These outcomes were also supported by the present study: in fact, lower pain and swelling were apparent in osteotomies performed by an ultrasonic device, probably due to the precision of the cut of the ultrasonic instruments.

In a previous study, significantly higher evidence of microcrack density after drill implant site preparation was found, in contrast to the ultrasonic preparation approach [25]. Moreover, extensive microcracks were not evident in the crestal or in trabecular part of the preparations adopting a piezoelectric approach and the few that were found were localized at the implant site. In contrast, the number of microfractures were higher in the cortical portion when compared to the cancellous bone area in the sites prepared using the drill technique, in which the incision line simply produces a local compression and a shift of the trabecular into the adjacent implant site. Therefore, this outcome highlighted that the differences between the piezoelectric approach and the conventional drilling of the implant site were only situated in the cortical portion. These results confirmed the outcome of a previous investigation, in which fragments of cortical bone generated by the osteotomic cut were studied. The tissue damage was oriented to the removal by osteoclastic activity, although knowledge about the cellular patterns and the molecule process involved in this field are scarce [36,37]. Moreover, in a rat model it has been demonstrated that bone microfractures are related to an increase in osteocyte cell apoptosis; this has been associated to the cortical portion resorption produced by the mechanical loading related to the micro-damage [38]. Piezoelectric implant bed preparation increased the secondary stability at 2 and 3 months compared to the conventional drilling approach in a study in which the dental fixture survival rate was 97.5% in the conventional drill group and 100% in the piezoelectric study group [39]. Also, a recent study showed that implant stability was slightly increased in cases of osteotomy performed by the use of piezoelectric bed preparation [40].

These results suggest that osteocyte death by apoptosis has a key function as a determinant in the induction of pain and swelling related to the bone damage.

Comparative histological studies [24] between piezoelectric devices, saws and drills have reported the superiority of the ultrasonic technique in order to preserve and protect the local anatomic structures, and consequently, an increased healing process. An important feature of the ultrasonic approach is the high clinical manageability, which simplifies the creation of a straight osteotomic shape without a specific training period for the surgeon [41,42]. The ultrasonic cavitation process is able to remove the debris of the intervention, with a morphology of the cut surface that appears porous, extremely clean and allows a fast link to fibrin [43,44]. The present research showed that implant bed preparation with ultrasonic tips had predictable outcomes with a high success rate. Further studies are needed to evaluate whether implant osseointegration periods could be shortened with ultrasonic implant bed preparation. However, the differences in pain and swelling observed in the present study are minimal and probably they have little relevance in clinical practice. They could be related to the prescribed anti-inflammatories. For this reason, it would be interesting to replicate this study without the use of the anti-inflammatory drug, and probably the difference in pain and swelling would be greater. Contrarily, the investigation outcome also showed that these positive effects are accompanied by a significative increase in the operating time for site preparation. In fact, the results of the present study indicate that the benefits in the implant bed preparation are also associated with an increased operative time of 372% compared with the drill group. The study also showed that there is no difference in crestal bone remodeling. Within the limitations of this study, the osteotomies performed by an ultrasonic device produced less pain and swelling, which is clinically advantageous; however, there was a significant increase in the operating time with a piezoelectric device approach.

## 4. Materials and Methods

### 4.1. Patient Selection

The study protocol was performed following the guidelines of the Helsinki Declaration (revised version of Tokyo Declaration in 2004) and Good Clinical Practice Principles. The trial was approved by the Inter Institutional Ethics Committee of Faculdade Ingá, UNINGÁ, PR, Brazil, N 89018318.2.0000.5220 in the period between September 2014 and August 2016.

The patients recruited for the clinical trial received treatment at the Department of Oral Implantology, Center for Advanced Studies, Dental Research Division, UNINGÁ—Cachoeiro de Itapemirim, Brazil.

The inclusion criteria of the study protocol comprised the complete or partially edentulous mandible of the candidates with a unilateral or bilateral absence of the teeth. The exclusion criteria comprised severe systemic diseases, head and neck radiotherapy and/or chemotherapy, uncompensated diabetes or periodontitis and smoking.

A total of fifty candidates (29 women and 21 men, non-smokers, mean age 52 years, range 41–63 years) with no relevant medical history were included in the clinical randomized trial. All candidates were elected to receive at least two implants in the posterior mandible and scheduled for fixed prosthesis or crown restoration. Preoperatively, the subjects received extensive information about the surgical treatment in order to obtain their complete cooperation during the surgery and the follow-up phase, and all candidates signed a written informed consent form. At the first visit, all candidates received a clinical and occlusal examination, and orthopantomography radiographs (OPT) were taken for diagnostic evaluation. Also, a Cone Beam Computer Tomography scan of the subjects (CBCT) (Vatech Ipax 3D PCH-6500, Fort Lee, NJ, USA) was performed to assist in the planning of the surgical procedure and to evaluate any clinically relevant evidence in terms of bone thickness and height.

### 4.2. Randomization and Study Design

The experimental implant sites were allotted into two different study groups, control (A) or test (B), with 25 sites each, by a computer-generated table, which was prepared using a balanced, randomly permuted implant site approach. A free random sampling and random assignment application was used (Research Randomizer Version 4.0, Geoffrey C. Urbaniak and Scott Plous). The primary predictor variable was the implant site preparation type. In Group A, conventional rotary instruments were utilized, and in Group B, the ultrasonic device was utilized for the preparation of the implant bed. All the implants were performed by three implant surgeons (AS, MM, PS) experienced in performing implant site preparation with piezosurgery devices. All the implants were placed in non-pathologic native bone sites. One kind of implant fixture was used for both groups: acid etched and only cylindrical implants were used (Isomed Albignasego, Padova, Italy) to better define and restrict variables in this study. The implant length used was 10 mm and 4.1 mm in width.

### 4.3. Surgical Procedures

At six days before the surgery, the candidates received an accurate professional oral hygiene prophylaxis and were instructed to perform correct domiciliary oral care, improved with 0.2% chlorhexidine digluconate mouth rinse solution for 2 min (Curaden Healthcare S.p.A., Saronno, Italy) after tooth brushing, once a day.

The subjects’ mouths were rinsed with chlorhexidine digluconate solution 0.2%for 2 min prior to the surgery. Then, local anesthesia of the tissues was performed by Articaine Pierrel (Pierrel S.p.A, Milan, Italy) with an epinephrine ratio of 1:100.000. Midcrestal incisions were made in order to expose the bone crest of the surgical sites and osteotomies of the implant beds were created. After implant bed preparation and implant placement the flaps were repositioned and sutured carefully with Polimid 4.0 (Sweden & Martina, Italy). Amoxicillin (1 g 2 times per day) was prescribed for one week as an antibiotic prophylaxis. Candidates who were allergic to the penicillin antibiotic were prescribed Clindamycin 300 mg twice a day for 6 days. An analgesic medication by Ibuprofen 600 mg was prescribed 2 h after the surgical procedure and every 6 h after the treatment in order to reduce the symptoms, continuing for 2–3 days following surgery. Those protocols were to guarantee the benefit of the medicines prior to the wearing off of the effect of the local anesthetic, though this made it more difficult to calculate the perioperative pain.

All implant placements were performed in all cases without any augmentation procedures. A total of 50 implant sites were prepared: half (n = 25) used conventional drills and half (n = 25) used an ultrasonic device (Surgysonic, Esacrom, Imola, Italy). In group A, the implant bed was performed using a first-round bur, followed by 2–3 and 3.9-mm-diameter burs (Bone System, Milano, Italy) at a speed of 400 rpm in accordance with the company’s instructions. In group B, the implant bed was prepared using an ultrasonic piezoelectric device, mounted with a cylindrical tip. The manufacturer protocols define the use of the following tip sequence: ES012X, ES052XG, ES040, ES041, ES043, and ES044 for Surgysonic protocol, this tip was without an internal water-cooling system. The implant surgeries were performed in two stages, according to the standard protocols of the surgery following an implant insertion torque of 30–40 N/cm. The duration of each operation was recorded in minutes, i.e., the duration of the flap incision until suturing; changing the drill or tip were excluded from the duration time of each operation. The total time of the action of the drill or tip was counted. The time necessary to complete the implant bed preparation (expressed in minutes) was calculated, starting from drill or tip bone contact to the end, after removal of the last drills/tip from the implant site. The surgical preparation was rinsed with sterile double-distilled water by the perfusion from the tip of the piezoelectric device after every use of the tips. After the use of the last ultrasonic tip, the implants were positioned by the surgical micromotor without the final single-use drill of the implant manufacturer. All implants were placed in a crestal position.

The medicines were taken by the patients with soft foods and/or a large glass of fruit juice in the absence of any side effects like nausea or stomach upset. To help reduce any discomfort after surgery, application of an ice bag for 10 min was also prescribed to reduce or eliminate any swelling. No corticosteroids were prescribed. The suture removal was performed at one week after surgery. The subjects were visited for 6 days and the surgical follow-up visits were scheduled at 1, 2, 4, and 6 day intervals to evaluate the surgical healing processes. The recall program included assessment of the VAS and VRS.

### 4.4. Pain and Swelling Intensity Evaluation

The level of postoperative pain was evaluated and scored by means of a 100 mm VAS scale from 0 (no pain) to 100 (worst pain imaginable) at 1, 2, 4, 6 day intervals. The pain level was then classified into four different categories: No pain (0 on the VAS), mild (1–35 mm), mild to moderate (36–50 mm), moderate (51–75 mm), and severe (76–100 mm). Swelling was recorded and classified into four categories: number 1 stands for the absence of swelling, intra-oral swelling in the area of the surgery scored 2, any extra-oral swelling in the area of the surgery scored 3, an intense swelling exhibited by extra-oral swelling extending beyond in the area of the surgery scored 4. At each control the temperature was also measured and recorded. An independent operator assessed the level of the swelling and pain at 1, 2, 4 and 6 postoperative days. All experimental measurements were collected at the same time every day.

### 4.5. Crestal Bone Level Measurement

A three-dimensional radiographic tomography scan (CBCT) was performed to investigate the crestal bone tissue resorption. The marginal bone height was recorded at the distal, mesial, lingual and buccal sites of each implant position, by the software (Iluma DVT software, IMTEC Imaging), by measuring the distance from the implant shoulder to the first visible bone-to-implant contact, with the value being expressed in mm. The crestal bone resorption measurements were repeated in the mesial side and in the distal side for each implant, recorded from the coronal part of the dental fixture to the detectable margin of the alveolar bone immediately after implant placement, and at three months of healing before the healing screw placement.

### 4.6. Statistical Evaluation

The power analysis of the experimental design model was performed by a specific statistical software (http://clincalc.com/stats/samplesize.aspx) able to determine the optimal sample size of the implant bed sites needed to achieve the outcome of a statistical significance for quantitative analyses of pain score, swelling score, crestal bone resorption and quantization of time for implant bed preparation. A calculation model was adopted for dichotomous variables (yes/no effect) by putting the effect incidence at 20% for controls and 80% for treated, alpha was set at 0.05 and power at 90% associated with the null hypothesis that the population means of the two groups were equal. The optimal size of the sample for the investigation was 24 experimental sites for each study group.

Implant was used as the statistical unit and the statistical analysis of the data was performed by the use of Statview software (SAS Institute, Cary, NC, USA). Continuous variables are presented by means of number of observations, mean and standard deviation. The Shapiro-Wilk test was performed to evaluate the normality of the data distribution of the VAS pain score, swelling score, operative time of the surgery and crestal bone resorption. The study data were compared between groups by the Mann–Whitney test, VAS and swelling within groups were evaluated by the Friedman test where a *p* value < 0.05 was considered statistically significant.

## Figures and Tables

**Table 1:** Summary of recording of VAS score and swelling score at different times after surgery. The VAS pain score and swelling score (VRS) were significantly decreased in the ultrasonic surgery group.

Study Groups	Pain (VAS Score)				Swelling (VRS Score)			
Group A (Drill)	Day 1	Day 2	Day 4	Day 6	Day 1	Day 2	Day 4	Day 6
Mean Rate (SD)	16.33 ± 4.12	19.22 ± 2.22	0.92 ± 0.3	0.11 ± 0.02	1.9 ± 0.8	1.2 ± 0.4	0.36 ± 0.31	-
Group B (Ultrasonic)	Day 1	Day 2	Day 4	Day 6	Day 1	Day 2	Day 4	Day 6
Mean Rate (SD)	12.33 ± 2.32	15.32 ± 3.34	0.82 ± 0.01	0.1 ± 0.01	1.27 ± 0.6	0.9 ± 0.58	0.27 ± 0.14	-
*p* value	*p* = 0.003 (**)	*p* = 0.04 (*)	*p* = 0.66	*p* = 0.57	*p* = 0.046 (*)	*p* = 0.28	*p* = 0.8	

Note. ** *p* < 0.01, * *p* < 0.05.

**Table 2:** Summary of operative time of implant site preparation. The crestal bone resorption was also evaluated at 3 months.

Study Groups	Surgical Time			Crestal Bone Resorption
Group A (Drill)	Operative Duration	Drill Change	Total time drill change	At 3 months
Mean (SD)	2.5 ± 0.34 min	5.5 ± 2.1 s	21.5 ± 2.1 s	0.03 ± 0.001 mm
Group B (Ultrasonic)	Operative Duration	Drill Change	Total time drill change	At 3 months
Mean (SD)	10.5 ± 3.1 min	9.3 ± 3.2 s	63.5 ± 2.1 s	0.036 ± 0.001 mm
*p* value	*p* = 0.00002 (**)	*p* = 0.00023 (**)	*p* = 0.00006 (**)	*p* = 0.8

Note. ** *p* < 0.01.

## References

[1] Albrektsson T., Lekholm U. (1989). Osseointegration: Current state of the art. Dent. Clin. North Am..

[2] Isola G., Matarese G., Williams R.C., Siciliano V.I., Alibrandi A., Cordasco G., Ramaglia L. (2018). The effects of a desiccant agent in the treatment of chronic periodontitis: A randomized, controlled clinical trial. Clin. Oral Investig..

[3] Matarese G., Ramaglia L., Cicciù M., Cordasco G., Isola G. (2017). The Effects of diode laser therapy as an adjunct to scaling and root planing in the treatment of aggressive periodontitis: A 1-year randomized controlled clinical trial. Photomed. Laser Surg..

[4] Schmidt K.E., Auschill T.M., Sculean A., Arweiler N.B. (2018). Clinical evaluation of non-surgical cleaning modalities on titanium dental implants during maintenance care: A 1-year follow-up on prosthodontic superstructures. Clin. Oral Investig..

[5] Burgos P.M., Meirelles L., Sennerby L. (2010). Early bone formation in furrows at titanium implants. A study in the rabbit tibia. J. Osseointegration.

[6] Scarano A., Crocetta E., Quaranta A., Lorusso F. (2018). Influence of the thermal treatment to address a better osseointegration of Ti6Al4V dental implants: Histological and histomorphometrical study in a rabbit model. Biomed. Res. Int..

[7] Scarano A., Degidi M., Perrotti V., Degidi D., Piattelli A., Iezzi G. (2014). Experimental evaluation in rabbits of the effects of thread concavities in bone formation with different titanium implant surfaces. Clin. Implant Dent. Relat. Res..

[8] Scarano A., Perrotti V., Artese L., Degidi M., Degidi D., Piattelli A., Iezzi G. (2014). Blood vessels are concentrated within the implant surface concavities: A histologic study in rabbit tibia. Odontology.

[9] Scarano A., Carinci F., Quaranta A., Iezzi G., Piattelli M., Piattelli A. (2007). Correlation between implant stability quotient (ISQ) with clinical and histological aspects of dental implants removed for mobility. Int. J. Immunopathol. Pharmacol..

[10] Scarano A., Degidi M., Iezzi G., Petrone G., Piattelli A. (2006). Correlation between implant stability quotient and bone-implant contact: A retrospective histological and histomorphometrical study of seven titanium implants retrieved from humans. Clin. Implant Dent. Relat. Res..

[11] Salami A., Mora R., Dellepiane M. (2008). Piezosurgery in the exeresis of glomus tympanicum tumours. Eur Arch Otorhinolaryngol..

[12] Salami A., Dellepiane M., Proto E., Mora R. (2009). Piezosurgery in otologic surgery: Four years of experience. Otolaryngol. Head Neck Surg..

[13] Eriksson R.A., Albrektsson T., Magnusson B. (1984). Assessment of bone viability after heat trauma: A histological, histochemical and vital microscopic study in the rabbit. Scand. J. Plast. Reconstruct. Surg..

[14] Sharawy M., Misch C.E., Weller N., Tehemar S. (2002). Heat generation during implant drilling: The significance of motor speed. J. Oral Maxillofac. Surg..

[15] Scarano A., Piattelli A., Assenza B., Carinci F., Di Donato L., Romani G.L., Merla A. (2011). Infrared thermographic evaluation of temperature modifications induced during implant site preparation with cylindrical versus conical drills. Clin. Implant Dent. Relat. Res..

[16] Vercellotti T. (2000). Piezoelectric surgery in implantology: A case report--a new piezoelectric ridge expansion technique. Int. J. Periodontics Restorative Dent..

[17] De Campos J.R.M., Wolosker N., Yazbek G., Munia M.A., Kauffman P., Puech-Leao P., Jatene F.B. (2010). Comparison of pain severity following video-assisted thoracoscopic sympathectomy: Electric versus harmonic scalpels. Interact. Cardiovasc. Thorac. Surg..

[18] Preti G., Martinasso G., Peirone B., Navone R., Manzella C., Muzio G., Russo C., Canuto R.A., Schierano G. (2007). Cytokines and growth factors involved in the osseointegration of oral titanium implants positioned using piezoelectric bone surgery versus a drill technique: A pilot study in minipigs. J. Periodontol..

[19] Schwarz F., Olivier W., Herten M., Sager M., Chaker A., Becker J. (2007). Influence of implant bed preparation using an Er: YAG laser on the osseointegration of titanium implants: A histomorphometrical study in dogs. J. Oral Rehabil..

[20] Horton J.E., Tarpley T.M., Wood L.D. (1975). The healing of surgical defects in alveolar bone produced with ultrasonic instrumentation, chisel, and rotary bur. Oral Surg. Oral Med. Oral Pathol..

[21] Vercellotti T., Nevins M.L., Kim D.M., Nevins M., Wada K., Schenk R.K., Fiorellini J.P. (2005). Osseous response following resective therapy with piezosurgery. Int. J. Periodontics Restorative Dent..

[22] Chiriac G., Herten M., Schwarz F., Rothamel D., Becker J. (2005). Autogenous bone chips: Influence of a new piezoelectric device (Piezosurgery^®^) on chip morphology, cell viability and differentiation. J. Clin. Periodontal..

[23] Ramli R., Reher P., Harris M., Meghji S. (2009). The effect of ultrasound on angiogenesis: An in vivo study using the chick chorioallantoic membrane. Int. J. Oral Maxillofac. Implants.

[24] Landes C.A., Stübinger S., Rieger J., Williger B., Ha T.K.L., Sader R. (2008). Critical evaluation of piezoelectric osteotomy in orthognathic surgery: Operative technique, blood loss, time requirement, nerve and vessel integrity. J. Oral Maxillofac. Surg..

[25] Scarano A., Iezzi G., Perrotti V., Tetè S., Staiti G., Mortellaro C., Cappucci C. (2014). Ultrasonic versus drills implant site preparation: A histologic analysis in bovine ribs. J. Craniofac. Surg..

[26] Scarano A., Sinjari B., Murmura G., Lorusso F. (2017). Neurosensory disturbance of the inferior alveolar nerve after 3025 implant placements. Implant Den..

[27] Eltayeb A.S., Ahmad A.G. (2017). Piezosurgery: A safe technique for inferior alveolar nerve mobilization in surgical correction of hemimandibular hyperplasia—Review of the literature and case report. Int. J. Surg. Case Rep..

[28] Rude K., Svensson P., Starch-Jensen T. (2018). Neurosensory Disturbances after Bilateral Sagittal Split Osteotomy using Piezoelectric Surgery: A Systematic Review. J. Oral Maxillofac. Surg..

[29] Sirolli M., Mafra C.E.S., Santos R.A.B.D., Holzhausen L.S., Neto C., Batista J. (2016). Influence of piezosurgery on bone healing around titanium implants: A histological study in rats. Braz. Dent. J..

[30] Di Alberti L., Donnini F., Di Alberti C., Camerino M. (2010). A comparative study of bone densitometry during osseointegration: Piezoelectric surgery versus rotary protocols. Quintessence Int..

[31] Büchter A., Kleinheinz J., Wiesmann H.P., Kersken J., Nienkemper M., Weyhrother H.V., Meyer U. (2005). Biological and biomechanical evaluation of bone remodelling and implant stability after using an osteotome technique. Clin. Oral Implants Res..

[32] Shalabi M.M., Wolke J.G., De Ruijter A.J., Jansen J.A. (2007). Histological evaluation of oral implants inserted with different surgical techniques into the trabecular bone of goats. Clin. Oral Implants Res..

[33] Iyer S., Weiss C., Mehta A. (1997). Effects of drill speed on heat production and the rate and quality of bone formation in dental implant osteotomies. Part I: Relationship between drill speed and heat production. Int. J. Prosthodont..

[34] Schlegel K.A., Kloss F.R., Kessler P., Schultze-Mosgau S., Nkenke E., Wiltfang J. (2003). Bone conditioning to enhance implant osseointegration: An experimental study in pigs. Int. J. Oral Maxillofac. Implants.

[35] Salami A., Dellepiane M., Salzano F.A., Mora R. (2007). Piezosurgery in the excision of middle-ear tumors: Effects on mineralized and non-mineralized tissues. Med. Sci. Monit..

[36] Frost H.M. (1986). Bone microdamage: Factors that impair its repair. Current Concepts of Bone Fragility.

[37] Parfitt A.M. (2002). Targeted and nontargeted bone remodeling: Relationship to basic multicellular unit origination and progression. Bone.

[38] Noble B.S., Peet N., Stevens H.Y., Brabbs A., Mosley J.R., Reilly G.C., Reeve J., Skerry T.M., Lanyon L.E. (2003). Mechanical loading: Biphasic osteocyte survival and targeting of osteoclasts for bone destruction in rat cortical bone. Am. J. Physiol. Cell Physiol..

[39] García-Moreno S., González-Serrano J., López-Pintor R.M., Pardal-Peláez B., Hernández G., Martínez-González J.M. (2018). Implant stability using piezoelectric bone surgery compared with conventional drilling: A systematic review and meta-analysis. Int. J. Oral Maxillofac. Surg..

[40] Sendyk D.I., Oliveira N.K., Pannuti C.M., Naclério-Homem M. da G., Wennerberg A., Zindel Deboni M.C. (2018). Conventional drilling versus piezosurgery for implant site preparation: A meta-analysis. J. Oral Implantol..

[41] Salami A., Mora R., Dellepiane M., Crippa B., Santomauro V., Guastini L. (2010). Piezosurgery versus microdrill in intact canal wall mastoidectomy. Eur. Arch. Otorhinolaryngol..

[42] Salami A., Vercellotti T., Mora R., Dellepiane M. (2007). Piezoelectric bone surgery in otologic surgery. Otolaryngol. Head Neck Surg..

[43] Scarano A. (2017). Traditional Postextractive implant site preparation compared with pre-extractive interradicular implant bed preparation in the mandibular molar region, using an ultrasonic device: A randomized pilot study. Int. J. Oral Maxillofac. Implants.

[44] Carini F., Saggese V., Porcaro G., Baldoni M. (2014). Piezolelectric surgery in dentistry: A review. Minerva Stomatol..

